# Underlying Topography Inversion Using Dual Polarimetric TomoSAR

**DOI:** 10.3390/s21124117

**Published:** 2021-06-15

**Authors:** Xing Peng, Shilin Long, Youjun Wang, Qinghua Xie, Yanan Du, Xiong Pan

**Affiliations:** 1 School of Geography and Information Engineering, China University of Geosciences (Wuhan), Wuhan 430074, China; pengxing@cug.edu.cn (X.P.); lsl_cug@cug.edu.cn (S.L.); youjunwang@cug.edu.cn (Y.W.); xieqh@cug.edu.cn (Q.X.); 2 School of Geographical Sciences, Guangzhou University, Guangzhou 510006, China; yndu@gzhu.edu.cn

**Keywords:** dual polarization, SAR tomography (TomoSAR), underlying topography, beamforming, Capon, MUSIC

## Abstract

Underlying topography plays an important role in the national economic construction, military security, resource exploration and investigation. Since synthetic aperture radar tomography (TomoSAR) can achieve the three-dimensional imaging of forests, it has been widely used in underlying topography estimation. At present, there are two kinds of TomoSAR based on the applied datasets: single polarimetric TomoSAR (SP-TomoSAR) and fully polarimetric TomoSAR (FP-TomoSAR). However, SP-TomoSAR cannot obtain the underlying topography accurately due to the lack of enough observations. FP-TomoSAR can improve the estimation accuracy of underlying topography. However, it requires high-cost data acquisition for the large-scale application. Thus, this paper proposes the dual polarimetric TomoSAR (DP-TomoSAR) as another suitable candidate to estimate the underlying topography because of its wide swath and multiple polarimetric observations. Moreover, three frequently used spectral estimation algorithms, namely, Beamforming, Capon and MUSIC, are used in DP-TomoSAR. For validation, a series of simulated experiments was carried out, and the airborne P-band multiple polarimetric SAR data over the Lope, Gabon was also acquired to estimate the underlying topography. The results suggest that DP-TomoSAR in HH & HV combination is more suitable to estimate underlying topography over forest areas than other DP combinations. Moreover, the estimation accuracy of DP-TomoSAR is slightly lower than that of FP-TomoSAR but is higher than that of SP-TomoSAR.

## 1. Introduction

The digital terrain model (DTM) plays an important role in the national economic construction, military security, resource exploration and investigation and other fields [1,2,3]. However, over forest areas, the traditional aerial survey and optical remote sensing technique can only obtain forest surface elevation, rather than the true terrain elevation. The underlying topography is usually estimated by subtracting the average vegetation height from the forest surface elevation. This method has poor accuracy and low efficiency, particularly for large-scale (global, national and regional) underlying topography inversion [4,5].

Synthetic aperture radar tomography (TomoSAR) is a suitable candidate to solve this problem because of its three-dimensional imaging capability [6,7,8,9]. TomoSAR forms an additional aperture along the vertical direction, which can discriminate scatterers in different heights in the same resolution cell. In particular, the long wavelength (e.g., L and P-band) SAR system, compared with the short wavelength (e.g., X and C-band) SAR system, has a strong penetrating ability over forest areas [10,11,12,13]. It can penetrate the canopy to the ground, acquiring the scattering signal from the ground. Therefore, TomoSAR has been widely used in the inversion of underlying topography over forest areas.

Based on the available datasets, there are two kinds of TomoSAR to estimate the underlying topography: single polarimetric TomoSAR (SP-TomoSAR) and fully polarimetric TomoSAR (FP-TomoSAR). SP-TomoSAR primarily uses tomograms in HH polarization to estimate the ground height, as HH polarization is sensitive to ground scattering. Although the backscattering power of the HH polarization channel is primarily obtained from the ground, it still records the significant forest canopy scattering information by penetrating the forest canopy. Thus, with SP-TomoSAR, it is hard to accurately obtain the underlying topography [14,15,16,17]. Compared with the single polarimetric SAR, fully polarimetric SAR can distinguish different scattering mechanisms in the same resolution cell [13,18]. Consequently, FP TomoSAR can not only acquire scatterers’ heights, but also identify their scattering mechanisms [19,20]. This technique is helpful for the interpretation of tomograms and can improve the accuracy of underlying topography inversion [21]. However, the FP system also has some disadvantages compared with the dual polarimetric (DP) system, such as high pulse repetition frequency [22,23], narrow swath width [24,25,26], and complicated system design caused by additional channel gain [27]. In particular, as a special DP system, compact polarimetric SAR can simultaneously obtain a rich surface information and realize large width observation, which attracts more attention. This suggests that a DP system can better meet the demands of timely updating and complete coverage in large scale or global scale monitoring.

At present, to obtain the forest backscattering power along the vertical direction, a number of spectral estimation methods have been proposed, which can be divided into three kinds: nonparametric spectral estimation, parametric spectral estimation, and sparse spectral estimation. Among these methods, Beamforming [28,29], Capon [30] and MUSIC [31,32,33] are three frequently used estimators. These three methods have already been widely tested by single/fully polarimetric SAR data. However, it is still not yet fully clear how they perform in the dual polarimetric mode. The motivation of this research was, therefore, to fill this knowledge gap by evaluating the dual polarimetric TomSAR in underlying topography inversion. DP-TomoSAR combines the backscattering power of two different polarization channels to separate surface scattering contribution and forest canopy contribution. Beamforming, Capon and MUSIC were expanded in DP-TomoSAR. For validation, a series of simulated experiments were carried out, and the airborne P-band multiple polarimetric SAR data over the Lope, Gabon were also acquired to estimate the underlying topography. Moreover, the LiDAR data were applied to assess their performance under different polarimetric modes.

## 2. Materials and Methods

### 2.1. DP-TomoSAR Model

SAR sensors have observed the same target area for *N* times, and *N* complex images can be obtained after imaging. The backscattering signal acquired by each pixel on the *n*-th image can be given by [7]:

(1) $$g_{n}\left(x,r\right)=\underset{\;}{\int }\beta \left(x,r,z\right)\cdot \exp\left[j\frac{4\pi b_{⊥n}z}{\lambda r\sin\theta }]dz=\underset{\;}{\int }\beta \left(x,r,z\right)\cdot \exp[jk_{z}\left(n\right)\cdot z\right]dz$$

where $k_{z}\left(n\right)=4\pi b_{⊥n}/\lambda r\sin\theta$ is the vertical wavenumber of the *n*-th image relative to the master image. x, r and  z represent the azimuth direction, slant range direction and vertical direction, respectively. $g_{n}\left(x,r\right)$ indicates the pixel value at  (x,r) of the two-dimensional SAR complex image obtained from the *n*-th image, and β(x,r,z) indicates the continuous reflectivity equation. λ is the carrier wavelength; $b_{⊥n}$ represents the length of the vertical baseline between the *n*-th image and the master image, and θ is the incidence angle.

Similar to FP SAR, DP SAR can not only acquire scatterers’ heights, but also identify their scattering mechanisms. There are three polarimetric combinations, including HH & HV, HH & VV and VV & VH. Here, taking HH & HV as an example, the continuous reflectivity function is discretised along the vertical direction Z, and the dual polarimetric SAR tomography model can be written as follows:

(2) $$\left[\begin{array}{c} g_{{\mathrm{HH}}_{1}}\\ g_{{\mathrm{HV}}_{1}}\\ \vdots \;\\ \begin{array}{c} \begin{array}{c} g_{{\mathrm{HH}}_{N}}\\ g_{{\mathrm{HV}}_{N}} \end{array} \end{array} \end{array}\right]=\left[\begin{array}{ccc} k_{1}\exp\left(jk_{z}\left(1\right)z_{1}\right) & \cdots & k_{D}\exp\left(jk_{z}\left(1\right)z_{D}\right)\\ k_{1}\exp\left(jk_{z}\left(2\right)z_{1}\right) & \cdots & k_{D}\exp\left(jk_{z}\left(2\right)z_{D}\right)\\ \vdots & \vdots & \vdots \\ k_{1}\exp\left(jk_{z}\left(N\right)z_{1}\right) & \cdots & k_{D}\exp\left(jk_{z}\left(N\right)z_{D}\right) \end{array}\right]\left[\begin{array}{c} \beta_{1}\\ \beta_{2}\\ \vdots \\ \beta_{D} \end{array}\right]+\left[\begin{array}{c} e_{{\mathrm{HH}}_{1}}\\ e_{{\mathrm{HV}}_{1}}\\ \vdots \;\\ \begin{array}{c} \begin{array}{c} e_{{\mathrm{HH}}_{N}}\\ e_{{\mathrm{HV}}_{N}} \end{array} \end{array} \end{array}\right]$$

where $k_{d}=[k_{1},k_{2}{]}^{T},d=1,2,\ldots D\;$ is the polarization measurement vector of two different polarization channels at the position *d* along the vertical direction and D is the number of discretised sampling.

Multi-look processing is performed in TomoSAR processing, and Formula (2) is written in matrix form:

(3) $$G_{\mathrm{DP}}\left(l\right)=A\left(z,K\right)\beta \left(l\right)+e_{\mathrm{DP}}\left(l\right),l=1,2,3,\ldots ,L$$

In Equation (3), $G_{\mathrm{DP}}={\left[g_{{\mathrm{HH}}_{1}},g_{{\mathrm{HV}}_{1}},\ldots ,g_{{\mathrm{HH}}_{N}},g_{{\mathrm{HV}}_{N}}\right]}^{T}$ is the observation vector with *N* images in DP mode; $\beta ={\left[\beta_{1},\ldots ,\beta_{D}\right]}^{T}$ represents the unknown parameter to be solved; $e_{\mathrm{DP}}={\left[e_{{\mathrm{HH}}_{1}},e_{{\mathrm{HV}}_{1}},\ldots ,e_{{\mathrm{HH}}_{N}},e_{{\mathrm{HV}}_{N}}\right]}^{T}$ indicates the noise vector from *N* images in HH and HV channel, and L is the number of looks.

For the 2N×D dimensional observation matrix A(z,K), which is composed of *D* steering vector $a(z_{d},k_{d})$, the following equations are used:

(4) $$a\left(z_{d},k_{d}\right)=k_{d}⊗a\left(z_{d}\right)\;A(z,K)=[a(z_{1},k_{1}),\cdots ,a(z_{d},k_{d}),\cdots a(z_{D},k_{D})]$$

where $a\left(z_{d}\right)=\left[\exp\left(jk_{z}\left(1\right)z_{d}\right),\exp\left(jk_{z}\left(2\right)z_{d}\right)\ldots ,\exp\left(jk_{z}\left(N\right)z_{d}\right)\right]$, and $K=\left[k_{1},k_{2},\ldots ,k_{D}\right]$.

### 2.2. Classical Spectral Estimation Methods for DP-TomoSAR

At present, Beamforming, Capon and MUSIC are the three most common spectral estimation algorithms in the field of TomoSAR. Therefore, these three methods are used to explore the performance of DP-TomoSAR.

#### 2.2.1. DP-Beamforming Estimator

The classical Beamforming method is regarded as a finite impulse response filter [34]. This ‘filter’ allows the signal of a certain spatial frequency to pass through without distortion and attenuates the signal of other frequencies.

Following the derivation of the classical Beamforming algorithm, the DP-Beamforming estimator is derived as follows. The optimisation of dual polarization Beamforming is given by:

(5) $$\underset{h}{\min}h_{B}^{H}h_{B}\;s.t.\;h_{B}^{H}a\left(z_{d},k_{d}\right)=1$$

where $h_{B}$ is the filter of the dual polarization Beamforming algorithm and it can be expressed as:

(6) $$h_{B}=\frac{a\left(z_{d},k_{d}\right)}{a^{H}\left(z_{d},k_{d}\right)a\left(z_{d},k_{d}\right)}=\frac{a\left(z_{d},k_{d}\right)}{N}$$

The power of the filtered signal is as follows:

(7) $$E\text{\{}\beta_{D}\left(l\right)\beta_{D}^{H}\left(l\right)\text{\}}=\frac{a^{H}\left(z_{d},k_{d}\right)Ra\left(z_{d},k_{d}\right)}{N^{2}}$$

It can be maximised according to the polarization state $k_{d}$ by:

(8) $$\underset{|\left|k_{d}\right|{|}_{2}=1}{\max}\frac{a^{H}\left(z_{d},k_{d}\right)Ra\left(z_{d},k_{d}\right)}{N^{2}}$$

where R is the covariance matrix of DP-TomoSAR model.

For the convenience of calculation, the steering vector $a\left(z_{d},k_{d}\right)$ is written as:

(9) $$a\left(z_{d},k_{d}\right)=(I_{(2\times 2)}⊗a\left(z_{d}\right))k_{d}=B\left(z_{d}\right)k_{d}$$

Thus, the maximum problem in Equation (8) can be transformed into the maximum eigenvalue problem of the Hermitian matrix $B^{H}\left(z_{d}\right)RB\left(z_{d}\right)$:

(10) $$B^{H}\left(z_{d}\right)RB\left(z_{d}\right)k_{d\max}=\lambda_{\max}k_{d\max}$$

Finally, the power by the DP-Beamforming is given by:

(11) $${\hat{P}}_{\mathrm{BF}}\left(z_{d}\right)=\frac{\lambda_{\max}\left(B^{H}\left(z_{d}\right){\hat{R}}_{\mathrm{DP}}B\left(z_{d}\right)\right)}{N^{2}}$$

where $\lambda_{\max}\left(\cdot \right)$ denotes the maximum eigenvalue of the positive semidefinite matrix. ${\hat{R}}_{\mathrm{DP}}=\frac{1}{L}\overset{L}{\underset{l=1}{\sum }}G_{\mathrm{DP}}(l)G_{\mathrm{DP}}^{H}(l)$ is the sample covariance matrix.

#### 2.2.2. DP-Capon Estimator

Some shortcomings of conventional Beamforming have been solved by the Capon algorithm, such as low height resolution, irregular sampling and serious sidelobe [28]. Thus, the principle of the Capon estimator is similar to that of Beamforming.

The optimisation problem using DP-TomoSAR can be written as follows:

(12) $$\underset{h}{\min}h_{C}^{H}h_{C}\;s.t.\;h_{C}^{H}a\left(z_{d},k_{d}\right)=1$$

By using the quadratic minimisation theorem, the filter of dual polarization Capon $h_{C}$ can be obtained:

(13) $$h_{C}=\frac{R^{-1}a^{H}\left(z_{d},k_{d}\right)}{a^{H}\left(z_{d},k_{d}\right)R^{-1}\left(z_{d},k_{d}\right)}$$

Then, the filtered signal power can be expressed as follows:

(14) $$E\text{\{}\beta_{D}\left(l\right)\beta_{D}^{H}\left(l\right)\text{\}}=\frac{1}{a^{H}\left(z_{d},k_{d}\right)R^{-1}a\left(z_{d},k_{d}\right)}$$

Maximizing it as regards the polarizations $k_{d}$ yields:

(15) $$\underset{|\left|k_{d}\right|{|}_{2}=1}{\max}\frac{1}{a^{H}\left(z_{d},k_{d}\right)R^{-1}a\left(z_{d},k_{d}\right)}$$

Combined with Equation (9), the problem of finding the maximum can be transformed into the problem of finding the minimum eigenvalue of Hermitian matrix $B^{H}\left(z_{d}\right)R^{-1}B\left(z_{d}\right)$:

(16) $$B^{H}\left(z_{d}\right)R^{-1}B\left(z_{d}\right)k_{d\min}=\lambda_{\min}k_{d\min}$$

The power of DP-Capon can be obtained as follows:

(17) $${\hat{P}}_{\mathrm{CP}}\left(z_{d}\right)=\frac{1}{\lambda_{\min}\left(B^{H}\left(z_{d}\right){\hat{R}}_{\mathrm{DP}}{}^{-1}B\left(z_{d}\right)\right)}$$

where $\lambda_{\min}\left(\cdot \right)$ is the minimum eigenvalue operator.

#### 2.2.3. DP-MUSIC Estimator

The MUSIC estimator can identify multiple spatial signals. Specifically, the sample covariance matrix is decomposed into eigenvalues, and the eigenvalues are sorted in descending order [35]. When the signal-to-noise ratio (SNR) is high, the signal eigenvalue can be distinguished from the noise eigenvalue [36].

When the classical MUSIC algorithm is introduced to DP-TomoSAR, the true height values $z_{d}$ and their related reflection mechanisms $k_{d}$ are the solutions of:

(18) $$a^{H}\left(z_{d},k_{d}\right)GG^{H}a\left(z_{d},k_{d}\right)=0$$

The pseudospectrum of the dual polarization MUSIC algorithm is expressed as

(19) $${\hat{P}}_{\mathrm{MU}}\left(z_{d}\right)=\frac{1}{\lambda_{\min}\left(B^{H}\left(z_{d}\right){\hat{G}}_{\mathrm{DP}}{\hat{G}}_{\mathrm{DP}}^{H}B\left(z_{d}\right)\right)}$$

where $\lambda_{\min}$ is the minimum eigenvalue; ${\hat{G}}_{\mathrm{DP}}$ is the eigenvector matrix, which can be obtained from eigenvalue decomposition of the sample covariance matrix.

## 3. Numerical Simulation

**Figure 1:**
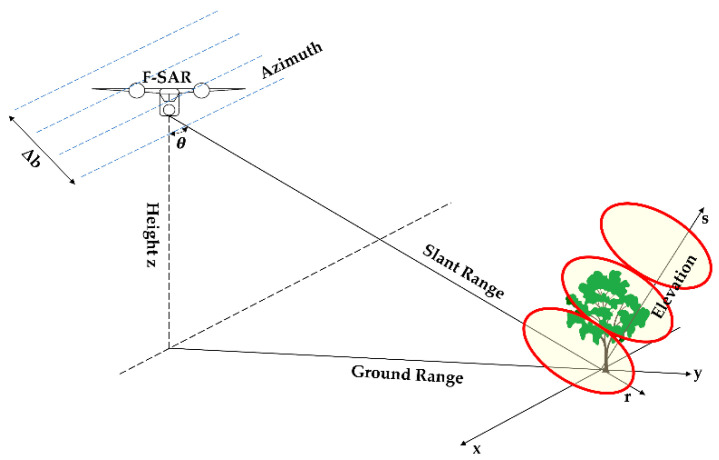
TomoSAR imaging under F-SAR system.

In general, the distribution of forest backscattered signals along the vertical direction primarily consists of two parts. The first part is the surface scattering signal with small angular spread distribution, which has a scattering centre located on the ground. The other part is the forest canopy scattering contribution with a large angle spread distribution [37]. We carried out two groups of simulation experiments to verify the feasibility and effectiveness of these three DP-TomoSAR algorithms. The parameters such as wavelength, slant range and baseline used in the experiment are shown in Table 1 and Table 2, which are obtained from the F-SAR airborne system, as seen in Figure 1.

### 3.1. Forest Vertical Profile Reconstruction

The first group of simulation experiments is conducted from three aspects to investigate the performance of forest vertical profile reconstruction:

(1) different SNR;
(2) different number of looks (N_obs);
(3) different height difference between the scattering centres of ground and canopy (Δh).

**Figure 2:**
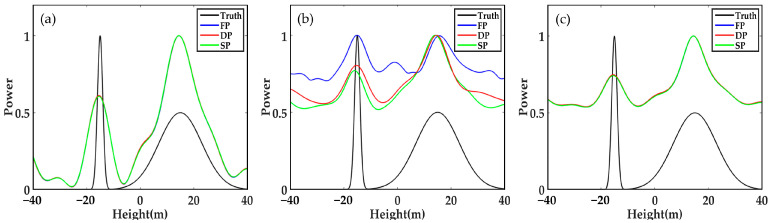
Forest backscattering profile reconstructed by different methods in SP, DP and FP mode when SNR = 20 dB, N_ob s = 100, and Δh = 30 m: (**a**) Beamforming, (**b**) Capon and (**c**) MUSIC.

As shown in Figure 2, the accuracy of FP-TomoSAR, DP-TomoSAR and SP-TomoSAR is equal under low noise, a large number of looks and large height difference between the ground and canopy scattering centres. They can all separate surface scattering contribution and canopy scattering contribution, obtaining the locations of ground and canopy scattering centres accurately.

**Figure 3:**
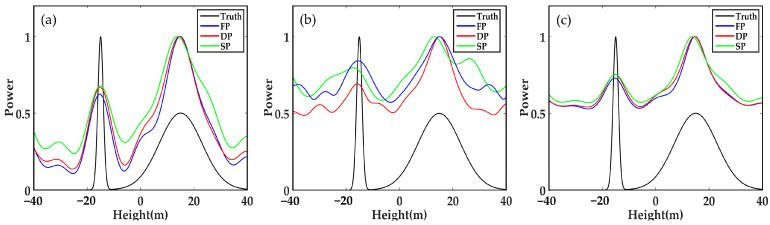
Forest backscattering profile reconstructed by different methods in SP, DP and FP mode when SNR = 5 dB, N_obs = 100, and Δh = 30 m: (**a**) Beamforming, (**b**) Capon and (**c**) MUSIC.

When decreasing SNR, as seen in Figure 3, the vertical profiles estimated by three methods based on FP data and DP data are also close to the true value, but the SP-TomoSAR estimation has some deviation from the true value. This finding indicates that in the case of strong noise, FP-TomoSAR and DP-TomoSAR have stronger abilities to obtain the ground scattering centres than SP-TomoSAR.

**Figure 4:**
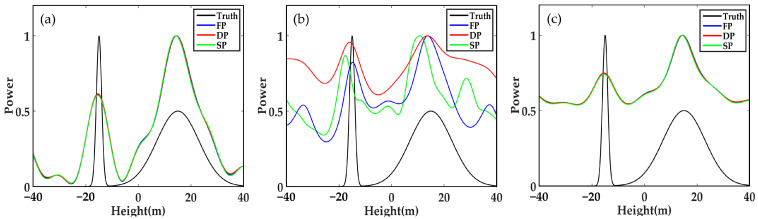
Forest backscattering profile reconstructed by different methods in SP, DP and FP mode when SNR = 20 dB, N_obs = 30, and Δh = 30 m: (**a**) Beamforming, (**b**) Capon and (**c**) MUSIC.

Then, we reduce the number of looks to a relatively low level. As shown in Figure 4, no evident effect is found on the Beamforming and MUSIC algorithm. However, the signal curve reconstructed by SP-Capon sharply deviates from the true value. Therefore, the results based on multi-polarization data have more advantages than those of SP-Capon algorithm.

**Figure 5:**
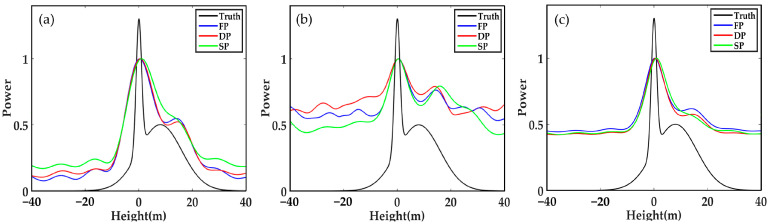
Forest backscattering profile reconstructed by different methods in SP, DP and FP mode when SNR = 20 dB, N_obs = 100, and Δh = 8 m: (**a**) Beamforming, (**b**) Capon and (**c**) MUSIC.

Finally, we discuss the influence of height difference between the ground scattering centre and the canopy scattering centre (Figure 5). Given the weak ability of the SP model to distinguish ground scattering and canopy scattering mechanisms, the detected ground scattering centres of the SP model have a right-wing shift compared with the results of the multi-polarization model.

### 3.2. Statisitical Analysis of the Scatterer Separation

**Figure 6:**
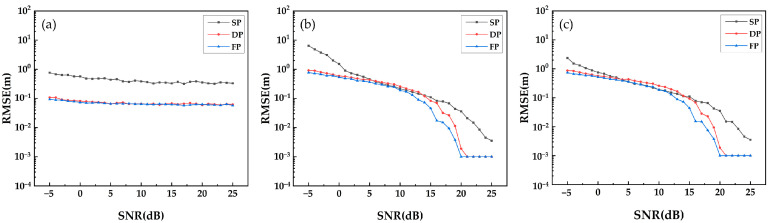
RMSE of underlying topography estimated by different methods in SP, DP and FP mode versus SNR: (**a**) Beamforming, (**b**) Capon and (**c**) MUSIC.

**Figure 7:**
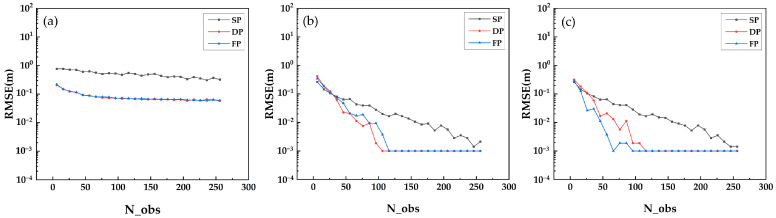
RMSE of underlying topography estimated by different methods in SP, DP and FP mode versus N_obs: (**a**) Beamforming, (**b**) Capon and (**c**) MUSIC.

**Figure 8:**
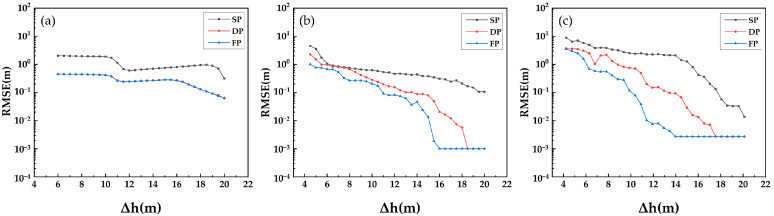
RMSE of underlying topography estimated by different methods in SP, DP and FP mode versus Δh: (**a**) Beamforming, (**b**) Capon and (**c**) MUSIC.

The second group of simulation experiments is conducted with a series of multiple repeated independent experiments to probe the resolution ability of three algorithms: Beamforming, Capon and MUSIC. As shown in Figure 6, Figure 7 and Figure 8, we calculate the RMSE of the estimated underlying topography versus SNR, N_obs and Δh.

Figure 6 shows that with the increase in SNR, the RMSE of underlying topography estimation indicates a convergent trend, and the single polarization data converge to a larger RMSE compared with multiple polarization data. As shown in Figure 7, the ability of DP-TomoSAR and FP-TomoSAR to detect the ground scattering centres is better than that of SP-TomoSAR. The convergent trend and convergent level of dual polarization are similar to those of the full polarization data. Figure 8 shows that the RMSE of underlying topography detected by DP-Beamforming and FP-Beamforming almost overlap, which are lower than the RMSE level of SP-Beamforming. The accuracy of dual polarization data based on Capon and MUSIC algorithms is between that of full polarization data and single polarization data.

Based on the two above-mentioned group experiments, under different conditions, the accuracy of underlying topography inversion obtained by DP-TomoSAR is better than that obtained by SP-TomoSAR, which is quite close to that of FP-TomoSAR. Therefore, using a dual polarimetric SAR tomography model to estimate the underlying topography is effective and feasible.

## 4. Experiments and Results

### 4.1. Study Area and Datasets

This paper uses 10 images at P-band obtained by the German F-SAR airborne system in the AfriSAR campaign to conduct experiments and verify the effectiveness of underlying topography inversion using DP-TomoSAR. Some basic parameters of the F-SAR system and baseline information are given in Table 1 and Table 2, respectively.

**Figure 9:**
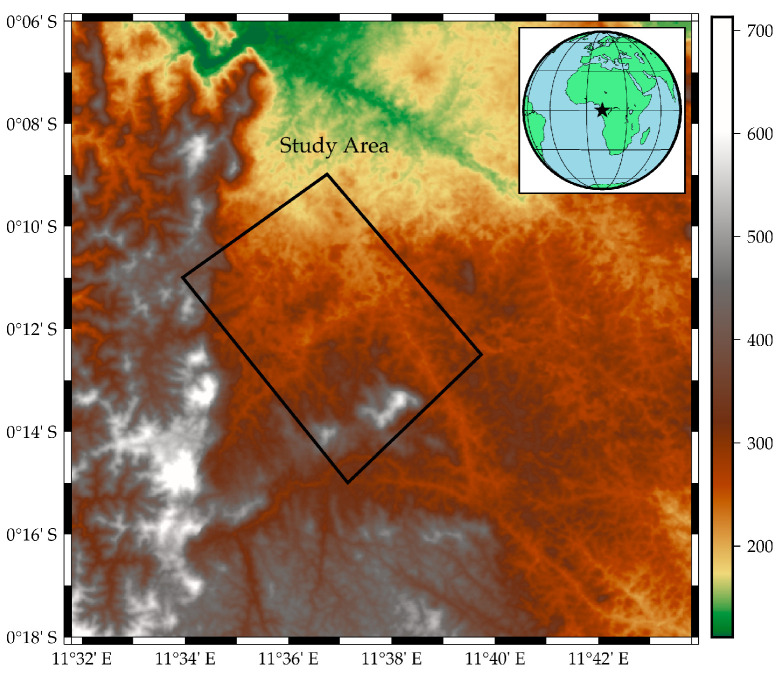
Location of the study area.

The data coverage is the Lope region, which is located in Gabon, Africa (Figure 9). Most of the experimental regions are hilly areas, primarily composed of savanna and dense forest areas [38], and the topography varies greatly, from 163 m to 583 m above sea level. In addition, the local slope of some areas can exceed 20°, and the maximum tree height is more than 50 m.

### 4.2. Results and Analysis

The tomogram of each pixel contains the backscattering power signals reconstructed by SAR tomography along the vertical direction. Hence, the analysis of the tomograms is a significant aspect for TomoSAR to obtain the 3D structure of forests. Tomograms are the key to underlying topography inversion.

**Figure 10:**
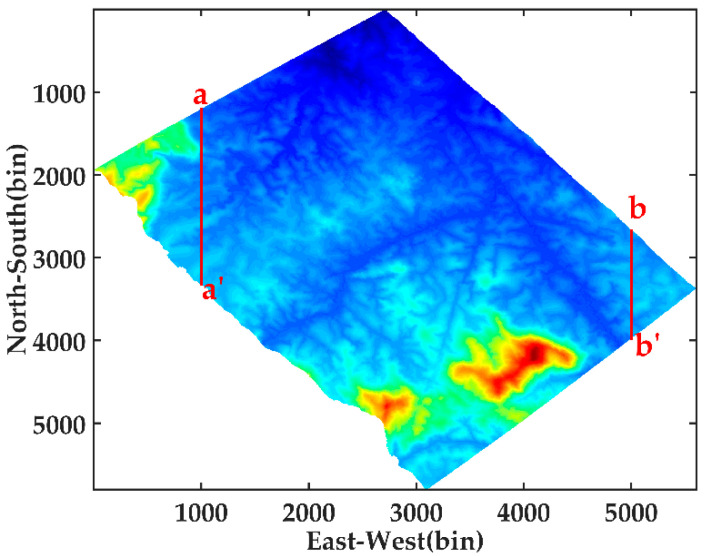
Two selected profiles (red solid lines aa′ and bb′) on LiDAR DTM image.

We select two profiles along the north–south direction (red solid lines as shown in Figure 10) under geographic coordinates to perform SAR tomography imaging. In general, for airborne SAR, some differences in the vertical wavenumber and noise level from the near range to the far range are observed. Thus, we consider line aa′ and line bb′ as a representation of profiles at the near range and far range, respectively. The coordinate system of the SAR data is inconsistent with that of the LiDAR data. The SAR coordinate system is geocoded to the same geographic coordinate as LiDAR for the convenience of the follow-up experiments. Moreover, the window size used to estimate covariance matrix is 31 × 31, and the LiDAR DTM data are sampled to the same size.

#### 4.2.1. Tomograms with Different Combinations

As mentioned in Section 2, the DP-TomoSAR model is composed of three polarisation combinations. At present, the suitable combination method for underlying topography inversion remains unknown. For the convenience of comparison, the LiDAR DTM is also projected onto the tomograms.

**Figure 11:**
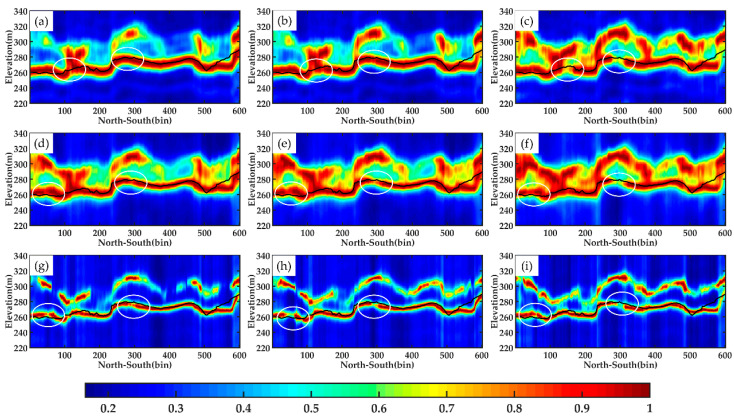
Tomograms of line aa′ estimated by different methods using each combination: (**a**–**c**) Beamforming, respectively, in HH & HV, HH & VV, and VV & VH; (**d**–**f**) Capon, respectively, in HH & HV, HH & VV, and VV & VH; (**g**–**i**) MUSIC, respectively, in HH & HV**,** HH & VV and VV & VH. The black solid lines represent LiDAR DTM, and the white circles mark the significant differences among tomograms.

For line aa′, Figure 11 shows the tomograms estimated by the three algorithms using different combinations of DP-TomoSAR. Profile aa′ is located in a position that has less noise disturbance in the whole image. The tomograms are relatively complete, and few discontinuous pixels are observed in the whole section. Amongst the three kinds of dual polarization combination, the combination of HH and HV polarizations has the best performance. Several evident discontinuities are found in the other two combinations, particularly in the VV&VH channel, as shown in white circles in these figures. Although the underlying topography information acquired by all combinations of DP-TomoSAR is consistent with LiDAR DTM data, we can still determine that for line aa′, the HH & HV is the most suitable dual polarization combination for underlying topography according to its most continuous tomograms.

**Figure 12:**
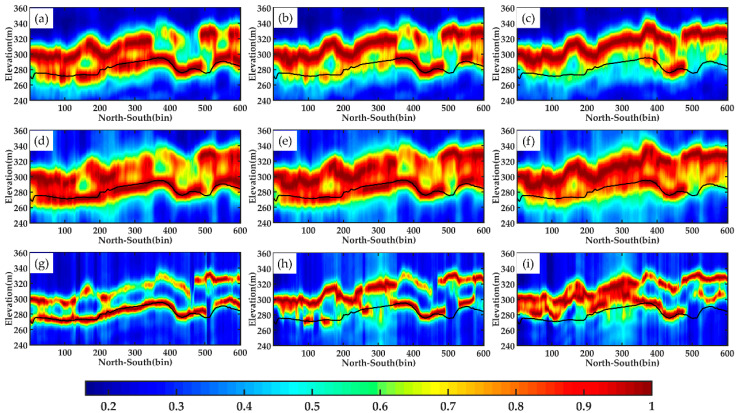
Tomograms of line bb′ estimated by different methods using each combination: (**a**–**c**) Beamforming, respectively, in HH & HV, HH & VV, and VV & VH; (**d**–**f**) Capon, respectively, in HH & HV, HH & VV, and VV & VH; (**g**–**i**) MUSIC, respectively, in HH & HV**,** HH & VV and VV & VH. The black solid lines represent LiDAR DTM.

Line bb′ is located in a position that has a high noise level in the whole image, which leads to many discontinuities in its tomograms. As shown in Figure 12, the differences amongst ground scattering estimated by different combinations are more significant than those of line aa′. In addition, based on this combination, the results of the HH & HV method have an advantage because each algorithm has obtained a relatively complete layer of underlying topography, which presents a similar trend to LiDAR data. Moreover, the subfigure (i) in Figure 12 indicates that the tomogram estimated by the MUSIC method based on VV&VH is greatly affected by the canopy information, resulting in a serious deviation between the terrain layer and LiDAR. Therefore, this combination is almost entirely not suitable for underlying topography inversion using DP-TomoSAR.

By analysing the imaging mechanism of each polarization channel, the causes of the above-mentioned phenomenon are summarised as follows. Firstly, the corresponding phase of the HH channel is sensitive to the ground scattering; therefore, the VV&VH combination obtained poor inversion results. Secondly, the phase centre of the VV channel is located in the area between the forest canopy and the surface, and the phase centre of the HV channel corresponds to the vegetation canopy. Hence, the combination of HH & HV is consistent with the scattering characteristics over forest covered areas. Theoretically, HH & HV is the optimal combination for retrieving underlying topography when using the DP-TomoSAR model.

Therefore, DP-TomoSAR combining two polarization channels, HH and HV, can effectively identify almost all the surface scattering contribution in the forest area. Using this to estimate the underlying topography is effective and feasible.

#### 4.2.2. Tomograms with Different Polarization Mode

**Figure 13:**
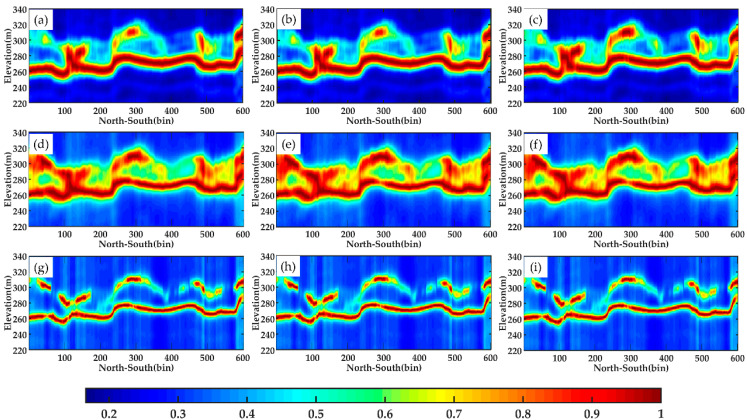
Tomograms of line aa′ estimated by different methods in SP, DP and FP mode: (**a**) Beamforming, SP (**b**) Beamforming, DP (**c**) Beamforming, FP (**d**) Capon, SP (**e**) Capon, DP (**f**) Capon, FP (**g**) MUSIC, SP (**h**) MUSIC, DP (**i**) MUSIC, FP.

**Figure 14:**
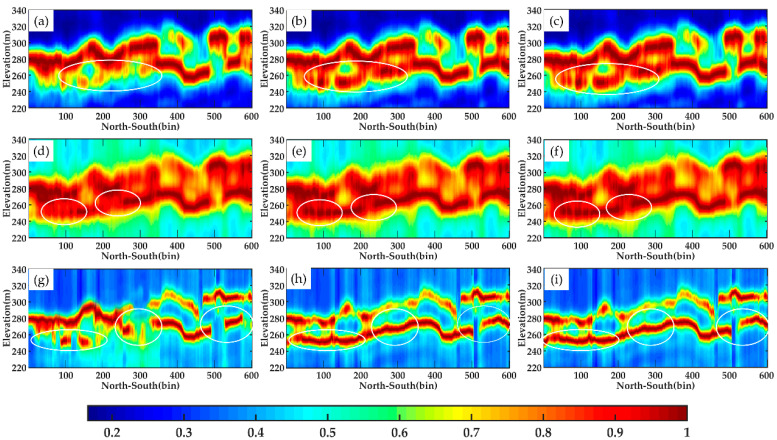
Tomograms of line aa′ estimated by different methods in SP, DP and FP mode: (**a**) Beamforming, SP (**b**) Beamforming, DP (**c**) Beamforming, FP (**d**) Capon, SP (**e**) Capon, DP (**f**) Capon, FP (**g**) MUSIC, SP (**h**) MUSIC, DP (**i**) MUSIC, FP. The white circles mark the significant differences among tomograms.

In verifying the performance of DP-TomoSAR, we use SP-TomoSAR and FP-TomoSAR to perform SAR tomography imaging on selected profiles line aa′ and line bb′ and obtain the tomograms (Figure 13 and Figure 14). The window size of the covariance estimation, height imaging range and height sampling interval is the same as those of dual polarization data.

As shown in the figures below, for line aa′, no significant difference is found amongst the single polarization tomograms, dual polarization tomograms and full polarization tomograms estimated by Beamforming, Capon and MUSIC. All the three estimators can detect the ground scattering centres well, which have a good agreement with the LiDAR DTM data. However, based on the tomograms of line bb′, the SP-TomoSAR could not obtain continuous ground scattering centres (particularly the white circles in Figure 14), whereas DP-TomoSAR and FP-TomoSAR can obtain the height of ground corresponding to the profile, probably because the line bb′ profile is located at the far range in the SAR coordinate system. Furthermore, the quality of the data is greatly affected by noise. In this paper, the simulation experiments in Section 3 have proven that the accuracy of DP-TomoSAR and FP-TomoSAR is higher than that of SP-TomoSAR when the noise is strong, and based on the real data experiments, the inversion results of line bb′ also confirm this point.

## 5. Discussion

**Figure 15:**
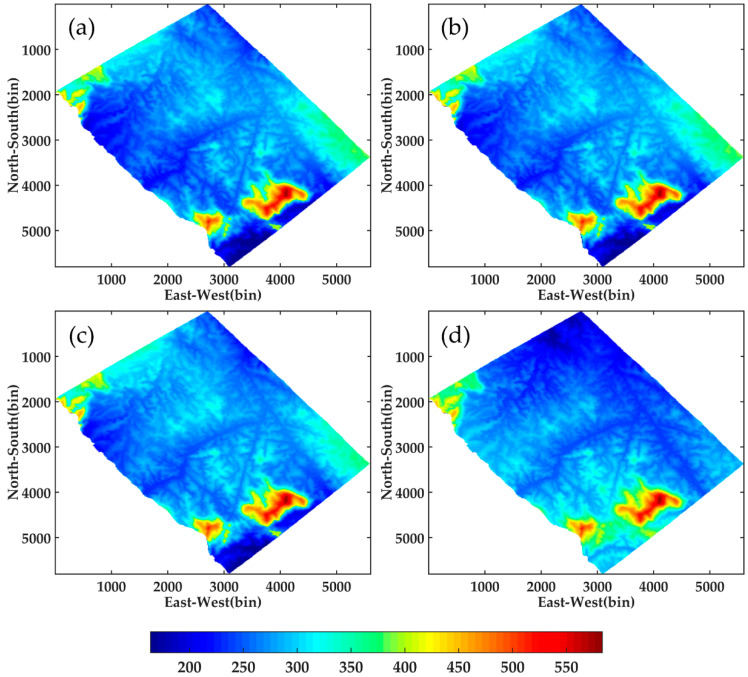
Underlying topography obtained by different methods of the study area: (**a**) DP-Beamforming (**b**) DP-Capon (**c**) DP-MUSIC and (**d**) LiDAR.

Beamforming, Capon and MUSIC algorithms are used in this paper to perform 3D focusing of SP, DP and FP data for the whole study area. The underlying topography under the same geographic coordinate system as the LiDAR DTM estimated by DP-Beamforming, DP-Capon and DP-MUSIC is shown in Figure 15.

As shown in the figure below, the underlying topography obtained by different DP-TomoSAR algorithms has a certain deviation from LiDAR DTM. However, they maintain a high consistency in texture features. This finding may be due to the large elevation span of the study area, in which the height above sea level of the terrain is from 163 m to 583 m. In addition, point cloud data at the edge of the image obtained by LiDAR are lacking, owing to the irregularity of the study area, which affects the accuracy of DTM generated by interpolating point cloud data to a certain extent. Thus, the results obtained by LiDAR data and TomoSAR data are different in the edges and corners of the image.

Moreover, RMSEs among different algorithms using SP-TomoSAR, DP-TomoSAR and FP-TomoSAR with respect to LiDAR DTM are calculated. The results are listed in Table 3, which makes a comprehensive analysis on the performance of three algorithms under different polarization mode.

For the Beamforming estimator, the RMSEs of underlying topography are 8.07, 8.25 and 9.24 m for full polarization, dual polarization and single polarization data, respectively. The result of DP-Beamforming is only 0.18 m higher than that of FP data. However, the difference between DP and SP data reached nearly 1 m. Thus, the multi-polarization mode can enhance the inversion accuracy of the Beamforming method. For the Capon algorithm, the RMSEs estimated by FP-TomoSAR, DP-TomoSAR and SP-TomoSAR are 7.92, 8.09 and 9.20 m, respectively. Based on the above-mentioned observation, the RMSE of SP-Capon exceeds those of DP-Capon and FP-Capon by a margin of more than 1 m. Similar to the estimation of the Beamforming method, the RMSE of DP-Capon is closer to that of FP-Capon and is smaller than that of SP-Capon. This result suggests that the underlying topography retrieved by DP-Capon is reliable. Furthermore, the RMSEs of MUSIC algorithm are 8.01, 8.17 and 9.21 m. Similar to the other two algorithms, the MUSIC method also shows certain advantages in dual-polarization mode.

In particular, no matter which polarization mode is used, Capon has the best performance in underlying topography inversion. Compared with DP-Beamforming, DP-Capon and DP-MUSIC have obtained a wider range of improvement in inversion accuracy, possibly because Capon has improved the resolution along the vertical direction of Beamforming algorithm. Moreover, the Capon method can suit the noise and interference signal in factual image data because of its adaptability; thus, this method can obtain a precise inversion result. As for MUSIC method, in this paper, we set the hyperparameter as 2 according to the canopy volume scattering and the surface scattering phase centres in a pixel over forest areas. However, the forest canopy includes several scatterers; thus, the number of scatterers in a resolution unit is still a relatively complex research object. Therefore, the performance of MUSIC algorithm is inferior to Capon in underlying topography inversion.

The accuracy of the results retrieved by all the three algorithms using DP-TomoSAR and FP-TomoSAR is close, and both of them are superior to those of SP-TomoSAR. This result provides a strong theoretical and data support for the potential application of the dual polarization model in underlying topography.

For spaceborne SAR systems, the dual polarization system is easy to maintain. Moreover, DP-TomoSAR has a higher resolution, wider swath and more data acquisition modes. Thus, it has great importance in optimising working modes of spaceborne SAR systems and estimating the large-scale underlying topography. In the future, DP-TomoSAR may be a primary method to obtain large-scale or global-scale underlying topography with spaceborne SAR data.

## 6. Conclusions

This paper primarily explores the effectiveness of DP-TomoSAR to estimate the underlying topography and selects three common spectral estimation algorithms (Beamforming, Capon and MUSIC) in TomoSAR to estimate the underlying topography based on dual polarization data.

Firstly, the ability of DP-TomoSAR to detect ground scattering centres is evaluated on the simulation data. We found that when the SNR is lower, or the difference between surface scattering centres and canopy scattering centres is smaller, the inversion results of Beamforming, Capon and MUSIC algorithms based on DP-TomoSAR remarkably improved compared with SP-TomoSAR. Furthermore, the accuracy of the Capon algorithm based on dual polarization data is evidently higher than that of single polarization data when the number of multi-looks is fewer. In addition, the inversion results of DP-TomoSAR are highly consistent with those of FP-TomoSAR.

Secondly, we use 10 images at the P-band obtained by AfriSAR campaign in 2016 to estimate underlying topography. We identify the combination mode of dual polarization that performs best in retrieving underlying topography. Consequently, the DP-TomoSAR model composed of the HH and HV channels can obtain the most complete tomograms. Moreover, tomograms of the selected profiles aa′ and bb′ based on SP and FP data are used as a control group to evaluate the performance of DP-TomoSAR. The results indicate that in terms of tomograms, the inversion effect and precision of DP-TomoSAR is reliable.

Finally, 3D focusing is performed under different polarization data modes in the whole research area. Compared with the DTM obtained by LiDAR, the RMSEs estimated by the Beamforming, Capon and MUSIC algorithms based on DP data are 8.25, 8.09 and 8.17 m, respectively. The RMSEs of underlying topography based on the FP data are 8.07, 7.92 and 8.01 m for the three estimators, and those of the SP data are 9.24, 9.20 and 9.18 m. All the above analyses show that the ability of FP-TomoSAR is slightly better than DP-TomoSAR in retrieving underlying topography. However, they are significantly better than that of single polarization data.

The dual TomoSAR model can be an important method to meet the demand of large-scale underlying topography inversion.

## Figures and Tables

**Table 1:** Parameters of TomoSAR data at the P-band obtained from F-SAR.

Wavelength (m)	Polarization Mode	Platform Height (m)	Range of Incidence Angle (°)	Resolution of Range Direction (m)	Resolution of Azimuth Direction (m)
0.6890	HH + HV + VV	6096	20−50	3.84	2

**Table 2:** Baseline information of TomoSAR data in the Lope region.

Identifier	Obtaining Date	Baseline (m)
FL06-PS02	10 February 2016	0
FL06-PS03		10
FL06-PS04		20
FL06-PS05		40
FL06-PS06		60
FL06-PS07		80
FL06-PS08		−20
FL06-PS09		−40
FL06-PS10		−60
FL06-PS11		−80

**Table 3:** RMSE of underlying topography estimated by different algorithms using SP-TomoSAR, DP-TomoSAR and FP-TomoSAR.

Method	Data Type	RMSE (m)
Beamforming	SP	9.24
	DP	8.25
	FP	8.07
Capon	SP	9.20
	DP	8.09
	FP	7.92
MUSIC	SP	9.18
	DP	8.17
	FP	8.01

## Data Availability

Not applicable.
